# Extracts from the Records of the Boston Society for Medical Observation

**Published:** 1856-01

**Authors:** S. P. Sprague


### EXTRACTS FROM THE RECORDS OF THE BOSTON SOCIETY FOR MEDICAL OBSERVATION.

BY S. P. SPRAGUE, M.D., SECRETARY.

Dr. Cabot exhibited to the Society pathalogical specimens from a young man 17 years of age, who had necrosis of a portion of the superior maxilla. Two months ago, the patient had the right lateral, and both central incisors of the upper jaw filled, and into the former, some preparation was introduced for the purpose of killing the nerve. One month since, he first had pain and soreness in and about the lateral incisor, which very soon extended forward to the median line, and backward as far as the place from which the first molar had been removed, six months previously. The cheek swelled so much that it was impossible to open the right eye. At the same time, some swelling appeared about the palatal and alveolar processes of the right superior maxilla, and has continued to increase gradually to the present time.

Three weeks ago, an abscess pointed just above the lateral incisor ; it was opened, and discharged a considerable quantity of offensive pus. Even then, all of the teeth of the right half of the upper jaw, excepting the second molar, had become loose ; the lateral incisor so much so, that it was easily removed with the fingers.

From this date, (April 29) the pain has not been acute, but dull and heavy. The abscess then opened, has since filled twice, and discharged itself spontaneously. During the first week there was considerable fever. No dead bone has ever been thrown off.

May 19th.—The patient now presents himself with right cheek rather larger than the left. There is much swelling of the gum, extending along the roof of the mouth to the median line, and limited posteriorly by the second molar, which is perfectly firm. The part feels soft, and is movable ; the probe detects naked bone, and motion gives an indistinct crepitus, and there is fluctuation where an abscess before pointed.—Patient was etherized, and an incision was made along the edge of the alveolus, the knife being carried vertically. Four teeth were then extracted, and several irregular fragments of bone (one as large as a walnut) were removed. There was but little hemorrhage.

21st.—Patient has been very comfortable, and has not felt the slightest inconvenience from the operation.

Dr. Cabot also mentioned the case of a child, 12 years old, who had had one of the middle incisor teeth filled with an arsenical preparation for the destruction of the nerve. In a few hours the patient had great pain in the tooth, the face was swollen, and all the teeth became loose, so that they could be moved. Six weeks afterwards he came to Dr. Keep, who advised that they should be removed, which was done.—There was an opening in the cheek through which several pieces of the bone came away, and this continued six weeks before all the pieces were removed. Dr. K. has had several cases where teeth were filled with arsenic in which such results followed, and in the case of Dr. Cabot, Dr. K. thought they arose from the same cause.

Dr. Cabot thought it merely destroyed the vitality of the tooth, which then acting like a foreign body, produced ulceration of the alveolus and necrosis. The attachments around soft parts were perfectly healthy in both Dr. C. and Dr. K.'s cases.

Dr. Slade asked whether exposure to the fumes of phosphorus might not have been a cause of the disease. He had seen a case somewhat similar in a girl 18 years of age, who came to him for advice. An incisor of the upper jaw had been extracted. The cheek became swollen, and a small portion of bone followed the exit of the tooth. A molar next became loose, and was extracted, and thus the teeth continued to become loose, and were extracted one after another for two years. Now she has lost all the teeth up to the middle incisors. There has been a constant discharge of pus, and a probe passed into the cavity of the antrum. The girl had worked in a factory where friction matches were made, and he attributed the disease to phosphorus. In this case, also, the gums were perfectly healthy.

Dr. Cabot said there was no chance of poisoning by phosphorus in the cases he reported. One of the patients was a school girl, and the other a young man in a comfortable condition of life, not obliged to work for a living. Poisoning by phosphorus was slow, and required time.

Dr. Williams spoke of the practice of some dentists and surgeons, of trephining the alveola and removing the decayed bone.

Dr. Ellis inquired if it was a common practice to make use of arsenic for preserving teeth.

Dr. Cabot replied that it was not employed now by respectable dentists in this country. Teeth filled with arsenic, he was informed by Dr. Keep, all turned to a mahogany color.*—*Dental Recorder.*

[* We think that Drs. Cabot and Keep and the Boston Society for Medical observation are misinformed—or are we to infer that they mean that teeth are preserved and filled with arsenic. If so they are right and Dr. Keep's mahogany might be of a *very dark* color; but that respectable Dentists do not use arsenic to destroy the nerve is certainly an error —for we believe that it never was more used than at present and never so understandingly.—Ed.

